# EGFR as a Negative Regulatory Protein Adjusts the Activity and Mobility of NHE3 in the Cell Membrane of IPEC-J2 Cells With TGEV Infection

**DOI:** 10.3389/fmicb.2018.02734

**Published:** 2018-11-13

**Authors:** Zhou Yang, Ling Ran, Peng Yuan, Yang Yang, Kai Wang, Luyi Xie, Shilei Huang, Jia Liu, Zhenhui Song

**Affiliations:** Department of Veterinary Medicine, Southwest University, Chongqing, China

**Keywords:** transmissible gastroenteritis virus, NHE3, EGFR, infection, regulation

## Abstract

Transmissible gastroenteritis (TGE) has caused devastating economic losses to the swine industry worldwide, despite extensive research focusing on the pathogenesis of virus infection. The molecular pathogenic mechanism of TGEV-induced diarrhea in piglets is unknown. Intestinal diarrhea is closely related to the function of the Na^+^/H^+^ exchanger protein NHE3 in the brush border membrane of small intestine epithelial cells. The epidermal growth factor receptor (EGFR) may act to regulate NHE3 expression. In addition, EGFR may promote viral invasion of host cells. The present study aimed to determine whether NHE3 activity is regulated by altering EGFR expression to affect Na^+^ absorption in TGEV-infected intestinal epithelial cells. Porcine intestinal epithelial cells were used as models for TGEV infection. The results showed that Na^+^ absorption and NHE3 expression levels decreased in TGEV-infected cells. Proliferation of TGEV within IPEC-J2 cells could be inhibited by treatment with the EGFR inhibitor AG1478 and knockdown; resulting in recovery of Na^+^ absorption in TGEV infected cells and increasing the activity and expression of NHE3. Moreover, we demonstrated that NHE3 activity was regulated through the EGFR/ERK pathway. Importantly, NHE3 mobility on the plasma membrane of TGEV infected cells was significantly weaker than that in normal cells, and EGFR inhibition and knockdown recovered this mobility. Our research indicated that NHE3 activity was negatively regulated by EGFR in TGEV-infected intestinal epithelial cells.

## Introduction

Transmissible gastroenteritis (TGE), caused by transmissible gastroenteritis virus (TGEV), is one many gastrointestinal infections in piglets, characterized by diarrhea and high mortality (Wu et al., 2003). The villi of jejunum and ileum become shorter after TGEV infection, which destroys the absorption function of intestinal epithelial cells, affecting the transport of nutrients and electrolytes, and increasing the osmotic pressure in the intestinal lumen, finally leading to severe diarrhea and dehydration. Nowadays, TGEV is one of the most important diseases threatening pork production worldwide. Diarrhea in piglets is accompanied by malnutrition, low immunity, serious stunting of growth and development, and a significant reduction in survival, with a mortality rate of up to 100% (Chae et al., 2000; Kim and Chae, 2001). Therefore, a study on pathogenesis of diarrhea caused by TGEV in piglets would be beneficial to improve treatments and coping strategies to reduce the economic losses in the pig industry.

Transmissible gastroenteritis virus belongs to the coronavirus pathogens that naturally infect pigs (Goh et al., 2012). The virus is transmitted by the mouth–nose route, and appears in the epithelial cells of the intestinal mucosa and alveolar macrophages in piglets, resulting in extensive injury of the lungs under severe conditions (Sestak et al., 2008). The virus sequentially spreads in the nasal mucosa, lungs, digestive tract, and small intestine. The microvilli of the jejunum and ileum shrink sharply or even break down, decreasing the absorption area of the intestinal villi (Zhao et al., 2014). Cell membrane transporter and ion channel transporter activities in intestinal epithelial decrease, resulting in disrupted Na^+^ transport or an imbalance of absorption and secretion, causing substantial losses of nutrients and water-electrolytes in intestinal epithelial cells (Sun et al., 2014). As Na^+^ absorption decreases, the osmotic pressure of the intestine increases abnormally, resulting in malabsorption diarrhea.

Malabsorption diarrhea includes abnormal Na^+^ transporter activity in the brush border membrane of intestinal cells, which results in serious inhibition of Na^+^ absorption (Liu et al., 2014). Na^+^ absorption at the top of the brush border in intestinal epithelial cells of mammals is accomplished by two main pathways, including the functions of an Na^+^/H^+^ exchanger (NHE3) and an Na^+^/glucose co-transporter (SGLT1), in which NHE3 plays a dominant role (Coon et al., 2011). A large amount of NHE3 promotes Na^+^ absorption in the intestinal epithelium via an electroneutral pathway (Malakooti et al., 2014). Diarrhea is accompanied by disordered NHE3 activity.

NHE3 is a member of the third subtype of the Na^+^/H^+^ exchanger family, responsible for the stable absorption of water and Na^+^ in cells, and is highly expressed in the gastrointestinal tract and kidney (Zachos et al., 2005). NHE3 promotes the alternative transport of Na^+^/H^+^ between the small intestine and the apical membrane of the proximal tubule (Biemesderfer et al., 1993; Amemiya et al., 1995; Biemesderfer et al., 1997), and is responsible for the maintenance of Na^+^ resorption and the acid–base balance in mammals, mediating the exchange pathway of extracellular Na^+^ and intracellular H^+^ in normal physiology to promote the absorption of water in the intestinal tract (Yin et al., 2015). Knockout of the *Nhe3* gene in mice resulted in a reduction of NaHCO3 resorption by proximal tubules of up to 60% (Wang et al., 2004); thus, the main source of Na^+^/H^+^ absorption in the intestinal tract of mice was ablated (Schultheis et al., 1998; Gawenis et al., 2002).

Membrane proteins on mammalian cell membranes play important roles in the uptake of water-electrolytes and nutrients. The membrane proteins on the plasma membrane are mobile within the membrane (Lin and Nie, 1985), allowing them to diffuse laterally in the lipid bilayer and move to the microvillus of the brush border membrane to perform their functions in nutrients absorption and material transport. The dynamic transport of membrane protein NHE3 has been studied using fluorescence bleaching recovery (FRAP) technology, which showed that the lysophosphatidic acid (LPA)/LPA5R signaling pathway, mediated by the epidermal growth factor receptor (EGFR), is involved in the regulation of NHE3 activity in microvilli. LPA, as an inflammatory factor, directly induces intestinal anti-secretion, and intensively stimulated NHE3 activity to inhibit secretory diarrhea induced by cholera. The FRAP results showed that LPA could increase NHE3 mobility in inflammatory bowel disease. The dynamic transport of NHE3 on intestinal microvillus was regulated by stimulating an increase in extracellular secretion (Lin et al., 2010).

To date, there have been many studies on vaccines and drugs targeted to TGEV in China; however, there have been fewer studies on the pathogenesis of TGEV, and the factors affecting diarrhea caused by TGEV in piglets remain unclear. Studies showed that diarrhea could decrease the activity and mobility of NHE3 in the intestinal microvillus (Cha et al., 2010; Lin et al., 2010), and the amount of NHE3 decreased rapidly. A few studies on the regulation of NHE3 activity have been performed under normal physiological conditions; however, the effects on the activity of NHE3 during diarrhea caused by TGEV infection have not been reported. EGFR may influence TGEV entrance, enhancing the ability of the virus to infect intestinal epithelial cells (Hu et al., 2016). In addition, EGFR is involved in the regulation of NHE3 activity during its dynamic transport. We hypothesized that in TGEV-infected cells, the dynamic transport of NHE3 would be regulated by TGEV infection. NHE3 mobility on the microvillus of the brush border membrane would be altered and NHE3 activity would be inhibited, ultimately affecting Na^+^ absorption in intestinal epithelial cells. It is important to explore this possible regulatory mechanism of the pathogenesis of diarrhea caused by TGEV infection in piglets.

## Materials and Methods

### Cells, Viruses, and Reagents

Porcine jejunum intestinal cells (IPEC-J2) were grown at 37°C and 5% CO_2_ in Roswell Park Memorial Institute (RPMI) 1640 medium (Gibco, United States) supplemented with 4% fetal bovine serum (FBS, Gibco), respectively. IPEC-J2 cells were purchased from Shanghai Zishi Biotechnology. The Miller strain of TGEV was preserved in our laboratory. We selected the tyrosine kinase inhibitor AG1478 as the inhibitor of EGFR, based on amino acid sequence of EGFR from NCBI.

### Experiment of Gene Silencing

Lentivival vectors (pLKO.1) purchased from Wuhan Miaoling Biotechnology designed to express short hairpin RNA (shRNA). shRNA lentiviral particles were used to designate EGFR (pLKO.1-EGFR-p-shRNA) for silencing of EGFR expression. pLKO.1-TRC was used to generate control lentivival. IPEC-J2 cells in TGEV-Infected groups and un-infected groups were prepared, respectively, to be transfected with the EGFR-specific shRNA plasmid using Lipofectamine^TM^ 3000 (Invitrogen, United States), according to the manufacturer’s instructions.

### Construction of the Expression Vector

To measure NHE3 mobility in IPEC-J2 cells, the coding sequence of NHE3 (GenBank ID: XM_021077062.1) was used. On the basis of no change occurring to the amino acid sequence of NHE3, the coding sequence was amplified as a 2,511 bp fragment, with Nhe I and Hind III restriction sites inserted at the 5′ and 3′ ends, respectively. The NHE3 gene fragment was synthesized by Wuhan Miaoling Biotechnology and then cloned into vector pEGFP-N3 vector (Clontech, Mountain View, CA, United States) to assemble the full-length pEGFP-NHE3 recombinant fluorescence plasmid for FRAP measurements.

### Transfection of the Recombinant Plasmid

IPEC-J2 cells were cultured on glass-bottomed 35-mm plastic culture dishes in RPMI 1640 medium (Gibco), supplemented with 5% FBS, 1% penicillin–streptomycin at 37°C in a 5% CO_2_. Cells were incubated in a 6-well plate for at least 24 h before transfection (60–70% confluency), and then incubated with the transfection complex containing 2.5 μg of pEGFP-NHE3 and pEGFP-N3 (Vector) in 125 μL of Opti-MEM medium mixed with Lipofectamine^TM^ 3000 (Invitrogen, United States), which was added to the cells in a dropwise manner, according to the manufacturer’s instructions. After observation under a fluorescence microscope, we determined the stable expression of the fluorescence protein. For FRAP detection, a premix comprising RPMI 1640 and 30 μM AG1478 was evenly covered on surface of the cells. After incubation for 24 h, the cells were infected with 0.1 multiplicity of infection (MOI) of TGEV per well in 6-well plates.

### TCID_50_ Analysis

TGEV-infected cells were collected 48 h after treatment with AG1478, subjected to three cycles of freezing and thawing, diluted sevenfold from 10^−1^ to 10^−7^ consistently, and added to 96-well plates. Each dilution was added to eight replicated wells. The method of Reed and Muench was then used to calculate TCID_50_ of the virus for the different groups.

### Flame Atomic Absorption Spectrometry

Samples to determine the intracellular and extracellular Na^+^ levels of IPEC-J2 were collected from the cell lysate after treatment with radioimmunoprecipitation assay (RIPA) solution and from the cell culture supernatant, respectively. Deionized water was used for all dilutions. A Na^+^ standard solution (10 μg/mL) was prepared by dissolving 1 mL of Na^+^ standard solution (1,000 μg/mL) in 100 mL of deionized water. The potassium (K) blank solution was prepared by dissolving 2.593 g of KNO3 in 50 mL of 5% HNO3, and then diluting to 500 mL with deionized water to get a 5% HNO_3_+K solution containing 2 mg/mL of K. The Na^+^ standard solution was then diluted as standard working solutions to 0.05, 0.1, 0.2, and 0.4 μg/mL. The intracellular and extracellular samples were diluted by 9.5 × 10^4^-fold and 6 × 10^3^-fold, respectively, to prepare for Na^+^ detection. The instrument conditions of the TAS-990 atomic absorption spectrometer were set as follows: Wavelength of 589 nm, negative high-voltage of 297 V, current of 2 mA, and a gas flow of 1,200 mL min^−1^, to measure the Na^+^ concentration of samples collected from different groups.

### Quantitative Real-Time Reverse Transcription Polymerase Chain Reaction (qRT-PCR)

Quantitative analysis of *NHE3* mRNA levels in TGEV-infected cells was performed using qRT-PCR with specific primers. Samples to be tested were collected from IPEC-J2 cells after TGEV infection for different times. Total RNA was extracted using the RNAiso plus reagent (Takara, Tokyo, Japan), and single-stranded cDNA was synthesized by reverse transcription using RNA PCR^TM^ Kit (AMV) Ver 3.0 Kit (Takara) according to the manufacturer’s instructions. Quantitative real-time PCR was then performed using SYBR Premix EX Taq II (Takara), with the following specific primers: NHE3 (Forward: 5′-GACCATCAAGCCTCTGGTGC-3′and Reverse: 5′-AATGTCCTCGATGGCCGAGA-3′), β-Actin (Forward: 5′-CTCTTCCAGCCCTCCTTCC-3′and Reverse: 5′-GGTCCTTGCGGATGTCG-3′), under the following conditions: 30 s at 95°C, and then 39 cycles of 5 s at 95°C, followed by 30 s at 60°C. Triplicate measurements were applied to calculate the average cycle threshold (Ct) for each individual test using the QuantStudio^TM^ 3 System software (Applied Biosystems, Carlsbad, CA, United States).

### Western Blot Analysis

Protein samples separated by SDS-PAGE were transferred to nitrocellulose membranes (Bio-Rad). The membranes were incubated with the following primary antibodies: rabbit polyclonal anti-NHE3 (Affinity), rabbit polyclonal anti-EGFR (Biorbyt), rabbit polyclonal anti-phospho-EGF (Tyr1068) (Cell Signaling), rabbit polyclonal anti-phospho-ERK1/2 (Thr202+Tyr204) (Bioss), rabbit polyclonal anti-β-tubulin (Proteintech), with goat anti-rabbit IgG (H+L) antibodies (Sangon Biotech) as the secondary antibody. Images of blots were obtained using a VILBER Fusion FX5 imaging system (VILBER) and the gray levels of all bands were analyzed.

### Fluorescence Recovery After Photobleaching (FRAP)

Fluorescence recovery after photobleaching was used to determine the lateral mobility of pEGFP-NHE3 at the apical domain of polarized IPEC-J2 cells. IPEC-J2 cells were cultured on glass-bottomed 35-mm plastic culture dishes in RPMI 1640 medium without phenol red. The cells were then transfected using Lipofectamine^TM^ 3000 with pEGFP-NHE3, and then infected with TGEV, during which time the cells were not exposed to serum. The glass-bottomed culture dish was then placed on the microscope stage for FRAP measurement. The region of interest (ROI) used to collect signal was located in a square of the apical domain (0.3 μm) of the target cell. The mobile fraction and diffusion rate were then calculated. High laser power (100% power, 100% transmission) was used for photobleaching. Before and after bleaching, we used 20% laser power with 1% transmission to measure the fluorescence. During the measurement, the 488 nm line of a 400 mW Kr/Ar laser was used. One image was collected every 60 s, and the bleaching and recovery were set with 10 time series. The images were saved after capturing of all the time series. The fluorescence intensity of the anchoring bleached region during the whole recovery process of fluorescence bleaching was detected and analyzed by the image analysis system of the Zen blue software (ZEISS, Germany). The percentage of maximal bleached fluorescence could be calculated from the recovery rates. The intensity ratio of the fluorescence intensity before bleaching (Fi), after bleaching (F0), and during full recovery (F∞) in the bleached region were compared, and the mobile and immobile fractions of the ROI were calculated using the equation: Mf = (F∞− F0)/(Fi − F0) × 100%. In each treatment group, the data from at least five cells were used for quantification and were measured repeatedly.

### Statistical Analyses

All results in the figures are presented as the mean ± the standard deviation (SD) from three independent experiments, and were analyzed using GraphPad Prism 6 software (GraphPad Inc.). For each assay, a *t*-test was used for statistical comparison and a *p*-value < 0.05 was considered statistically significant.

## Results

### NHE3 Expression Is Related to Na^+^ Absorption in TGEV-Infected Cells

**FIGURE 1:**
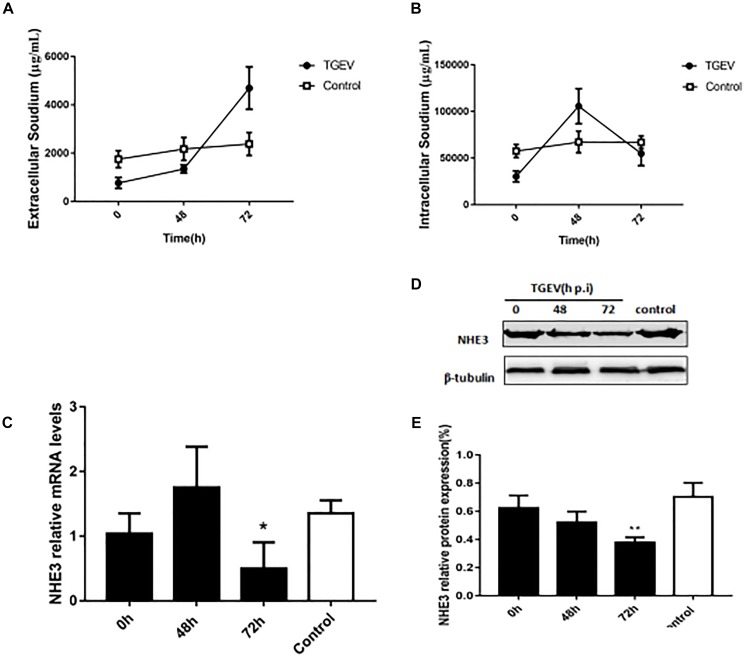
The effect of TGEV on Na^+^ concentration and the relative expression of NHE3 in IPEC-J2 cells. **(A,B)** Intra and extracellular Na^+^ concentration in cells infected with TGEV at 0, 48, and 72 h post infection. **(C)** NHE3 mRNA expression levels were detected using qRT-PCR and normalized by the β-actin mRNA level. **(D)** NHE3 protein levels were analyzed by Western blotting. **(E)** Grayscale analysis of NHE3 relative abundance changes. ^∗^0.01< *p* < 0.05, ^∗∗^*p* < 0.01.

Research on the mechanism of diarrhea has mainly focused on the physiological activity of Na^+^/H^+^ exchange proteins (Singh et al., 2014). Under normal physiological conditions, Na^+^/H^+^ exchanger NHE3 is generally responsible for the intake of Na^+^ in intestinal epithelial cells. Thus, cellular Na^+^ absorption is determined by the NHE3 activity. In mammals, the deletion of the NHE3 gene profoundly weakened Na^+^ absorption. Therefore, we examined the changes in the intra- and extracellular Na^+^ concentration in intestinal epithelial cells using flame atomic absorption spectrometry at 0, 48, and 72 h post-TGEV infection. Compared with the control group, the extracellular Na^+^ concentration of the TGEV-infected group continued to increase, reaching its highest levels at 72 h post-infection (p.i.) (Figure 1A). The intracellular Na^+^ concentration gradually increased up to 48 h p.i., and then began to decrease in the last period (Figure 1B). By contrast, the intra and extracellular Na^+^ levels in the control showed almost no change, suggesting that TGEV infection affected the intra- and extracellular Na^+^ concentration in piglet intestinal epithelial cells. Subsequently, we examined the expression level of the NHE3 gene (Figure 1C). In IPEC-J2 cells during 72 h of TGEV infection, after an initial increase in expression, the mRNA level of *NHE3* decreased to a level lower than that of the control. We then used Western blotting to study the effect of TGEV infection on level of the NHE3 protein in IPEC-J2 cell at 0, 48, and 72 h p.i. The results suggested that TGEV infection caused consistent downregulation of NHE3 levels, most significant at 72 h p.i. compared with 0 h p.i. (Figures 1D,E).

### TGEV Invasion Is Suppressed via Inhibition of EGFR

**FIGURE 2:**
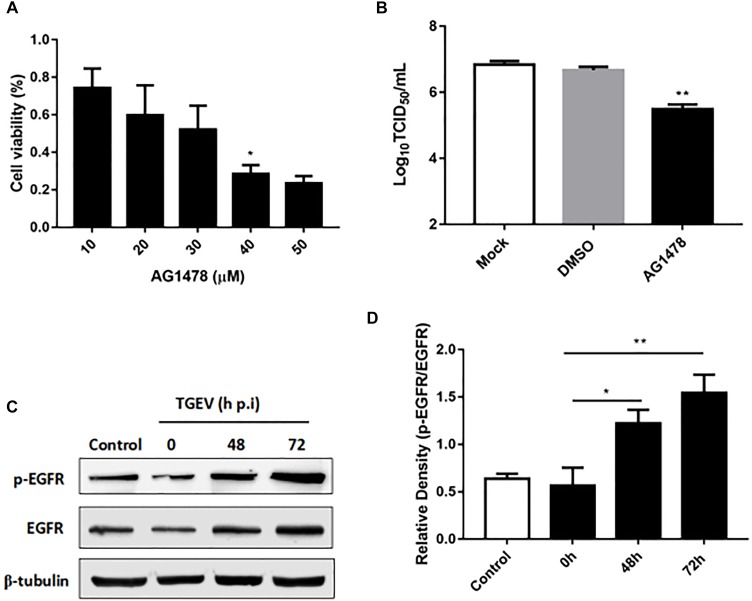
The role of EGFR in IPEC-J2 cells infected with TGEV. **(A)** MTT detection of cell viability after treatment with AG1478 at different concentrations in IPEC-J2 cells. **(B)** The propagation of TGEV in IPEC-J2 cells after treatment with 30 μM AG1478. **(C)** EGFR protein expression levels were analyzed by Western blotting. **(D)** Grayscale analysis for the level of phosphorylated EGFR in IPEC-J2 cells infected with TGEV at 0, 48, and 72 h post-infection. ^∗^0.01< p < 0.05, ^∗∗^*p* < 0.01.

Epidermal growth factor receptor is regarded as a transmembrane receptor for multiple viruses, including TGEV (Eppstein et al., 1985; Hu et al., 2016). To determine whether EGFR affects the process of TGEV invasion and infection of IPEC-J2 cells, we used Western blotting to assess its levels in TGEV-infected cells. We observed that the level of phosphorylated EGFR increased during TGEV infection (Figures 2C,D), especially at 72 h p.i. We then used AG1478 as an EGFR inhibitor to suppress EGFR in IPEC-J2 cells. The 3-(4,5-dimethylthiazol-2-yl)-2,5-diphenyltetrazolium bromide (MTT) assay showed that cell viability was not reduced after EGFR inhibition (Figure 2A), and that 30 μM was the best toxic dosage of AG1478 for IPEC-J2 cells. To study whether AG1478 could inhibit TGEV proliferation in cells, we used 30 μM AG1478 to treat cells for 24 h, followed by incubation with 10^4^ TCID_50_ TGEV for 48 h. The results showed that the average viral titer of the DMSO group (infected with TGEV after treatment with 30 μM DMSO) was 10^6.84^ TCID_50_/mL, and the titer of the AG1478 group (infected with TGEV after treatment with 30 μM AG1478) was 10^5.49^ TCID_50_/mL, representing a reduction of 93.3% compared with that of the DMSO group (Figure 2B). These data showed that proliferation of TGEV in IPEC-J2 cells could be reduced by inhibiting EGFR.

### Inhibition of EGFR Promotes Na^+^ Absorption in TGEV-Infected Cells

**FIGURE 3:**
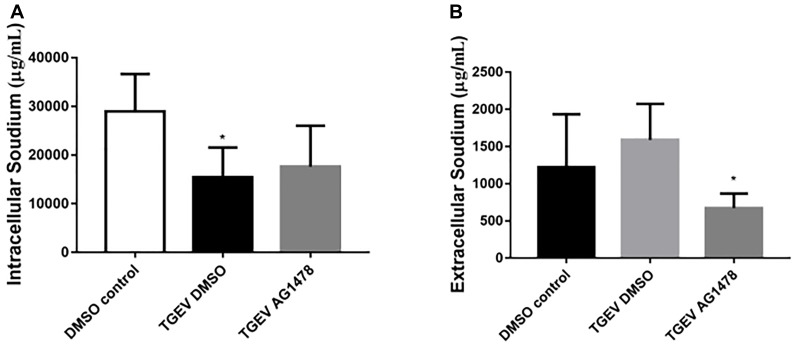
Effect of inhibition of EGFR on the intracellular and extracellular Na^+^ concentration in TGEV-infected cells. **(A)** Intracellular Na^+^ concentration in IPEC-J2 cells after treatment with 30 μM AG1478 or DMSO for 24 h; samples collected from the cell lysate were tested. **(B)** Extracellular Na^+^ concentration in IPEC-J2 cells after treatment with AG1478 or DMSO for 24 h; samples collected from the cell culture medium were tested. ^∗^0.01 < *p* < 0.05.

To further investigate the role of EGFR in Na^+^ absorption in TGEV-infected IPEC-J2 cells, we examined the effects of AG1478 treatment on Na^+^ absorption. As shown in Figure 3A, the intracellular Na^+^ concentration of IPEC-J2 cells was significantly increased by treatment with the EGFR inhibitor AG1478 during TGEV infection. By contrast, treatment with AG1478 significantly reduced the extracellular Na^+^ concentration (Figure 3B). These data showed that EGFR inhibition could restore the function of Na^+^ absorption in IPEC-J2 cells during TGEV infection.

### NHE3 Protein Levels Are Regulated Though the EGFR/ERK Pathway

**FIGURE 4:**
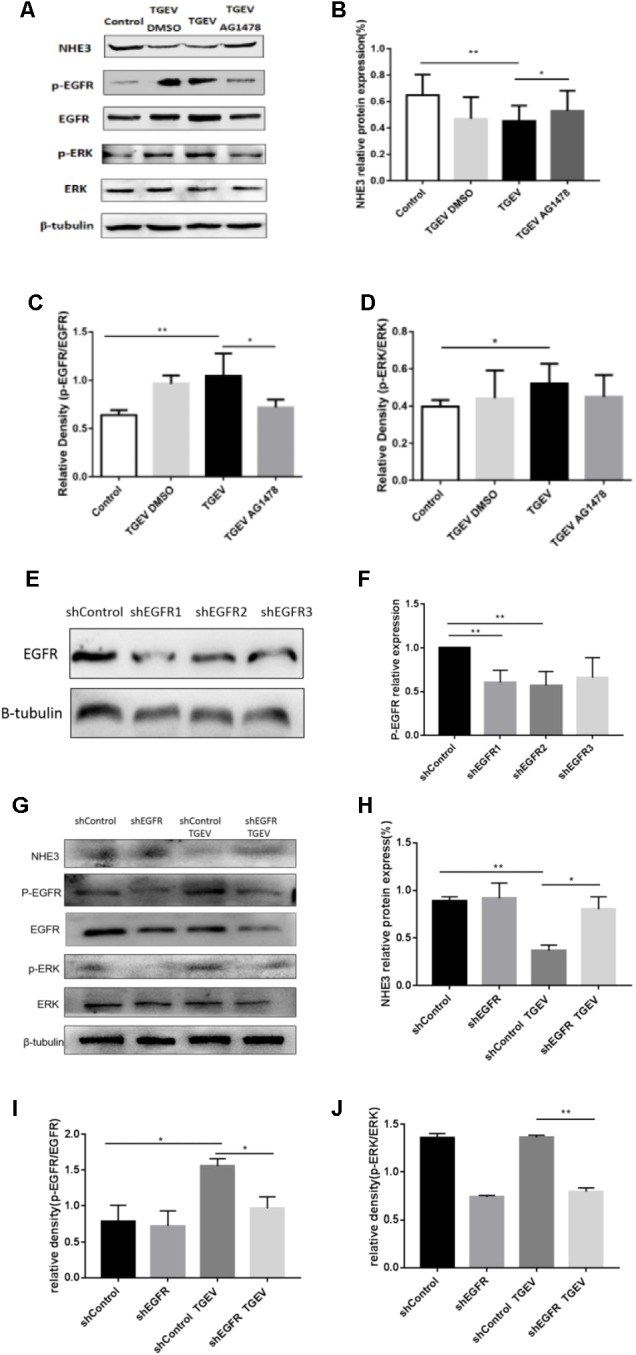
TGEV regulated the inhibition of NHE3 via the EGFR/ERK pathway. **(A)** IPEC-J2 cells in corresponding groups were treated with AG1478, protein levels of NHE3, p-EGFR, EGFR, p-ERK, and ERK in TGEV-infected cells or uninfected cells were analyzed by Western blotting using specific antibodies. **(B–D)** Grayscale analysis of the changes in NHE3, p-EGFR/EGFR, and p-ERK/ERK levels, as analyzed using the spass software. **(E,F)** Knockdown of EGFR expression in IPEC-J2 cells by short hairpin shEGFR lentivirus was decreased by 57–66% in shEGFR-infected cells compared with control cells. **(G)** IPEC-J2 cells in corresponding groups were treated with EGFR-specific shRNA, expression levels of p-EGFR, EGFR, p-ERK, ERK, and NHE3 proteins were evaluated by Western blotting analysis. **(H–J)** Grayscale analysis of the changes in p-EGFR/EGFR, p-ERK/ERK, and NHE3 levels, as analyzed using the spass software. Each experiment was performed in triplicate. ^∗^0.01< *p* < 0.05, ^∗∗^*p*< 0.01.

During viral invasion of host cells, the first step is to combine with receptors on the cell membrane, and then transfer signals into cells to stimulate a series of downstream signal cascade reactions (Homma and Yasui, 1968). The transmembrane receptor EGFR can promote virus invasion of host cells and activate downstream signaling pathways, of which the most common pathway is the MAPK signaling pathway. Diarrhea weakens the Na^+^ absorption function of intestinal epithelial cells. Na^+^ absorption is primarily mediated by NHE3 in the cell membrane. Therefore, we assessed whether the activity and expression of NHE3 was regulated via activation of the EGFR/ERK signaling pathway in IPEC-J2 cells during TGEV infection. As shown in Figure 4, we chose 30 μM AG1478 and DMSO to treat cells for 24 h, respectively, and then infected them with TGEV. Meanwhile, normal cells without any treatment were regarded as the control group. As shown in Figure 4B, the NHE3 protein level in the TGEV-infected group was significantly decreased compared with that in normal cells group, and its level in the TGEV plus AG1478 group was increased compared with that in the TGEV-infected group. Compared with the control group, the level of phosphorylated EGFR or ERK in the TGEV-infected group was significantly increased (Figure 4A). However, the level of p-EGFR in the TGEV plus AG1478 group was significantly lower than that in the TGEV-infected group (Figure 4C). The level of p-ERK was also downregulated (Figure 4D). We confirmed the knockdown efficiency of EGFR (Figures 4E,F). As well as the effect of EGFR inhibitor AG1478 on NHE3 via EGFR interference, transfection of PLKO.1-EGFR-p-shRNA in IPEC-J2 cells resulted in a significant increase in NHE3 activity, comparing with the TGEV-infected group (Figure 4H), suggesting that EGFR played a role in the regulation of NHE3 activity in IPEC-J2 cells, NHE3 activity could recover in TGEV-infected group after inhibition of EGFR. On the contrary, both phosphorylation levels of EGFR and ERK were significantly decreased compared with TGEV-infected group (Figures 4I,J), as well as knockdown of EGFR reduced the phosphorylation of EGFR and ERK in the TGEV-infected cells. These results suggested that the EGFR/ERK pathway is one of the pathways that regulate NHE3 protein activity in TGEV-infected cells.

### EGFR as a Crucial Protein Controls NHE3 Mobility in Cytomembrane

Stable mobility of the brush border membrane is very important for the uptake of Na^+^ and water-electrolytes in the intestinal epithelium. The membrane transporter NHE3 is mainly responsible for the electrically neutral translocation of Na^+^/H^+^ in intestinal epithelial cells, and changes in the transport capacity of Na^+^/H^+^ on the brush border membrane is determined by changes in NHE3 activity. In general, NHE3 on the plasma membrane diffuses to the top of the brush border to exert its function and stimulate the Na^+^ absorption in the intestinal epithelium. To further identify the dynamic changes of NHE3 on the brush border membrane caused by TGEV infection in small intestinal epithelial cells, NHE3 mobility on the membrane of TGEV-infected cells after treatment with EGFR was analyzed using FRAP.

**FIGURE 5:**
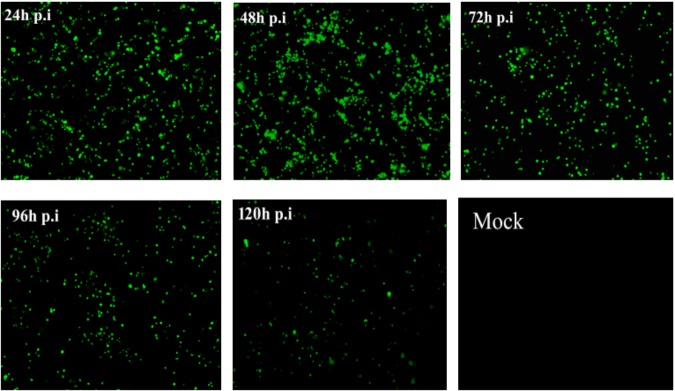
Fluorescence observation of IPEC-J2 cells transfected with pEGFP-NHE3 (10×). IPEC-J2 cells were transfected with recombinant vector (pEGFP-NHE3) for different times (24, 48, 72, and 120 h), and examined using fluorescence/phase-contrast microscopy.

The recombinant expression plasmid pEGFP-NHE3 was transfected into IPEC-J2 cells and cultured at 37°C with 5% CO_2_. The stable expression of the recombinant plasmid was observed under an inverted fluorescence microscope. The transient expression of green fluorescent protein (GFP) was observed under the control of a CMV promoter. Green fluorescence was produced using a 490 nm blue wavelength under the fluorescence microscope. The recombinant fluorescent-protein was expressed stably for 48 h and reached a peak at 24–120 h after transfection, as shown in Figure 5. Untransfected normal cells showed no fluorescence. The above results indicated that recombinant fluorescent plasmid pEGFP-NHE3 was transfected into IPEC-J2 cells and expressed the EGFP-NHE3 fusion protein successfully. The period of stable EGFP-NHE3 expression was about 5 days.

**FIGURE 6:**
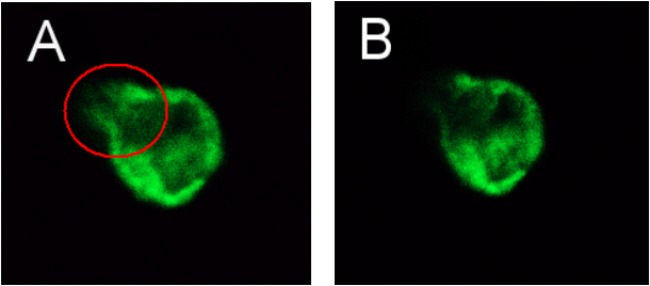
LSCM images captured before **(A)** and after **(B)** bleaching (63×/1.4 NA).

After transfection of the eukaryotic expression plasmid pEGFP-N3 into normal cells, this group was set as control vector group. FRAP analysis of eight cells was then used to determine the best time of fluorescence bleaching and recovery (Figure 6). After bleaching, the fluorescence intensity at the bleached region tended to be stable after 5 min. Figure 6 show the images captured before and after bleaching, and 5 min was chosen as the best time of bleaching. After bleaching using an intense laser, the change in fluorescence intensity recovery was observed in the bleached area. The fluorescence recovery curve showed that the fluorescence intensity of the bleached area gradually recovered with prolonged time, and the fluorescence intensity of the bleached region tended to stabilize after 8 min. Therefore, 8 min was chosen as the best time to detect the fluorescence recovery rate in each group. The recovery rate of fluorescence bleaching and mobile fractions were measured in the same time period.

**FIGURE 7:**
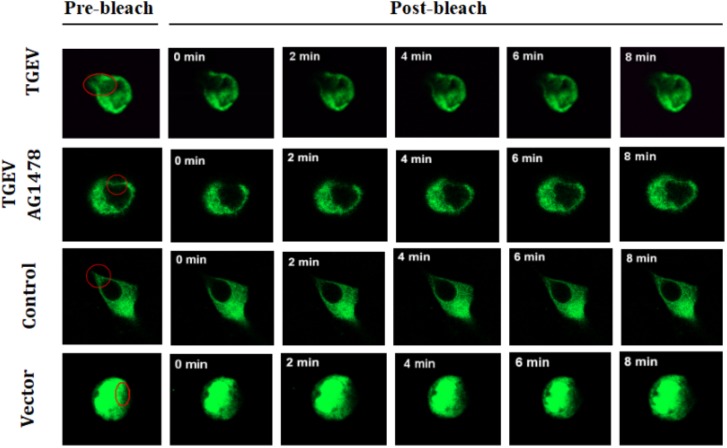
Dynamic changes of fluorescent molecules within bleached regions during fluorescence recovery after photobleaching (63×/1.4 NA). Representative images of FRAP experiments in different groups of IPEC-J2 cells with TGEV infection or not, and for those treated with AG1478. The first images on the left are from before photo-bleaching, subsequent images were taken immediately or at 0, 2, 4, 6, and 8 min after bleaching.

**FIGURE 8:**
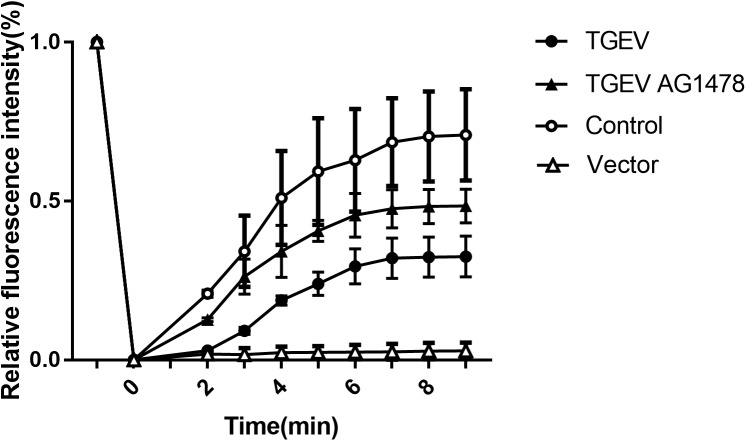
Fluorescence recovery rate on the surface of IPEC-J2 cell (2 mW, 63×/1.4 NA). FRAP recovery curves of pEGFP-NHE3 in different groups are shown. Error bars represent the mean ± SD.

Subsequently, the fluorescence images at five time points (0, 2, 4, 6, and 8 min) were selected to reflect the dynamic change of fluorescence bleaching and recovery in each group (Figure 7). The formula used to calculate the FRAP rate in each group was as follows: Kt = (Ft − F0)/(Fi − F0) × 100%, where Kt represents the fluorescence recovery rate at each time point, Ft stands for the fluorescence intensity after bleaching, F0 stands for the fluorescence at time 0, and Fi stands for the fluorescence intensity before bleaching. The fluorescence recovery curves according to the fluorescence recovery rate at different time points for different groups of cells were then drawn (Figure 8). The results showed that the fluorescence recovery rate of NHE3 in cells infected with TGEV was significantly lower than that in the control group. The fluorescence recovery rate of NHE3 in TGEV-infected cells after treatment with AG1478 was significantly higher than that in cells infected with TGEV, but was lower than that in the control group without TGEV infection. The fluorescence recovery rate in cells transfected with the empty vector was the lowest compared with the other three groups, showing almost no recovery.

**FIGURE 9:**
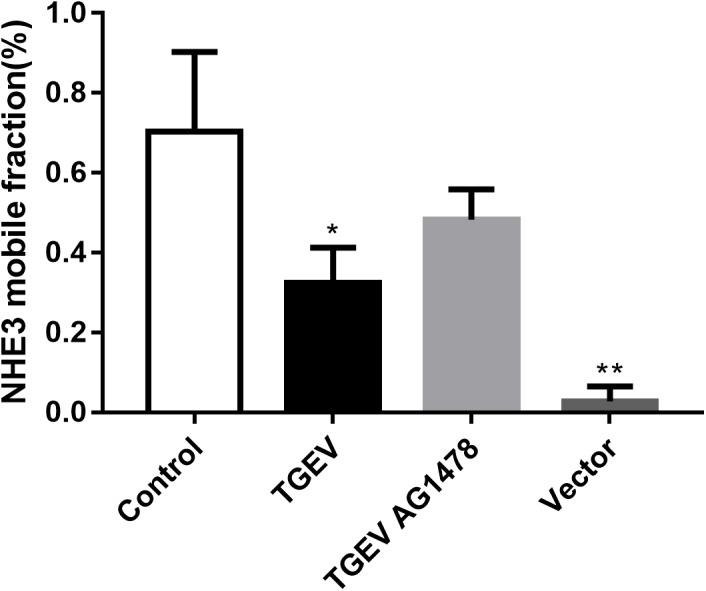
Analysis of NHE3 mobile fraction (2 mW, 63×/1.4 NA). Each test was replicated three times. FRAP data from eight different cells in each group were used for the calculation and analysis. ^∗^0.01< *p* < 0.05, ^∗∗^*p*< 0.01.

According to the analysis, the average value of stable fluorescence intensity restored before and after bleaching, and the mobile fraction of NHE3 in the four groups, was calculated using the following formula M_f_ = (F_∞_ − F_0_)/(F_i_ − F_0_) × 100%, where, F_∞_ was the fluorescence intensity returned to stability after bleaching, Fi was the fluorescence intensity before bleaching, and F0 was the fluorescence intensity at 0 min after bleaching. According to the analysis of the calculation results (Figure 9), the mobile fraction of the empty carrier group was reduced by 96% compared with the control group, and the difference was very significant (*p* < 0.01). The mobile fraction of NHE3 in TGEV-infected group decreased significantly by 54% (0.01 < *p* < 0.05). The mobile fraction of NHE3 in the TGEV plus AG1478 group increased by 33% compared with that in the TGEV group. The results showed that the non-quenched fluorescence molecules gradually diffused from the unbleached region to the bleached area with increasing time, and the fluorescence of the bleached region gradually recovered. Therefore, we believe that the membrane transporter NHE3 is mobile on the cell surface, and the different treatment groups had different NHE3 mobilities. NHE3 mobility on the plasma membrane was decreased in cells infected with TGEV compared with normal cells, and NHE3 mobility in cells infected with TGEV after treatment with AG1478 increased. These results indicated that decreased EGFR activity in intestinal epithelial cells was accompanied by increased NHE3 mobility after TGEV infection. Thus, EGFR negatively regulates NHE3 mobility on the plasma membrane in TGEV-infected cells.

## Discussion

Previous studies showed that the absorption of Na^+^ and water in the intestines dropped sharply and led to severe diarrhea in NHE3 deficient mice. The expression and activity of NHE3 were significantly inhibited under conditions of diarrhea and severe intestinal inflammation induced by cholera toxin (Coon et al., 2011; Yang et al., 2015). According to results of flame atomic absorption spectrometry, during TGEV infection, the intracellular Na^+^ concentration increased before 48 h p.i., but decreased from 48 to 72 h p.i. The extracellular Na^+^ concentration also increased, with a peak at 72 h p.i.

It is believed that the Na^+^/H^+^ exchanger protein NHE3 has two main functions. One is to transport extracellular Na^+^ into cells and the other is to secrete intracellular H^+^ to the extracellular area. Under normal physiological conditions, homeostasis is maintained between the intra and extra-cellular Na^+^ concentration. Na^+^/H^+^ exchanger 3 (NHE3) and Na-glucose cotransporter-2 can deliver Na^+^ from the extracellular medium into cells. At the same time, an intracellular Na–K pump will pump out the intracellular Na^+^, thus to maintaining a normal Na^+^ concentration gradient on both sides of the plasma membrane. Thus, the Na^+^ concentration in inside and outside of uninfected cells seemed to be stable, without any obvious increases or decreases, which was in accordance with the theoretical basis. By contrast, TGEV infection resulted in a decrease in Na^+^ absorption and reduced Na^+^ transportation from the outside to inside of cells by NHE3, leading to a gradual increase in the extracellular Na^+^ concentration and a decrease in the intracellular Na^+^ concentration. Sodium/glucose cotransporter 1 (SGLT1) also plays a role in intracellular Na^+^ absorption, acting as a Na^+^-glucose co-transporter, which transports Na^+^ across the plasma membrane of animal cells down an electrochemical gradient. Glucose is “dragged” into the cells against the concentration gradient. Meanwhile, SGLT1 can promote Na^+^ absorption and glucose uptake of epithelial cells. Research has shown that SGLT1 plays a critical role during the process of TGEV infection in cells. Dai et al. (2016) reported that SGLT1 protein levels were upregulated and glucose uptake in the small intestine was enhanced in the early stage of TGEV infection of IPEC-J2 cells. However, later, the glucose intake of the intestinal epithelium decreased (Dai et al., 2016).

Our results showed that the intracellular Na^+^ concentration in TGEV-infected cells increased before 48 h p.i. One of the likely explanations was that in IPEC-J2 cells with TGEV infection, SGLT1 could transport extracellular Na^+^ into the cells down a concentration gradient during the early phase of infection with TGEV, to increase intracellular glucose intake. The subsequent decrease in the intracellular Na^+^ concentration likely reflected the continuous infection with TGEV into the cells promoted by the increase of intracellular glucose intake, resulting in atrophy and rupture of intestinal epithelial microvillus. As the absorption area of intestinal villi decreases (Zhao et al., 2014), the quantity of NHE3 and mobility of in the brush border would also decrease. As the NHE3-mediated Na^+^ transportation decreased, the intracellular Na^+^ decreased and the extracellular Na^+^ concentration increased.

We next investigated whether TGEV infection regulated the activity, abundance, and mobility of NHE3 through EGFR in IPEC-J2 cells. The spike protein (S) of TGEV combines with EGFR to activate the downstream PI3K signaling pathway, allowing TGEV to invade small intestinal epithelial cells (Dai et al., 2016). Our results showed that the levels of p-EGFR and p-ERK were significantly upregulated in porcine intestinal epithelial cells after TGEV infection. In addition, the EGFR inhibitor AG1478 and kockdown of EGFR downregulated the levels of p-EGFR and p-ERK. Compared with TGEV-infected cells, the NHE3 levels in cells with TGEV infection after using EGFR inhibition and shRNA lentiviral particles were upregulated. These results showed that TGEV infection regulated NHE3 via the EGFR/ERK signaling pathway, and there was a negative correlation between EGFR and NHE3. EGFR and ERK are activated by TGEV infection in IPEC-J2 cells; however, AG1478 and PLKO.1-EGFR-p-shRNA could significantly reduce the proliferation of TGEV by inhibiting EGFR. Inhibition of EGFR reduced the damage caused by TGEV to intestinal epithelial cells, ameliorated the loss of intracellular Na^+^ caused by TGEV infection, restored the normal function of membrane transporter NHE3, and promoted the transport of extracellular Na^+^ into cells. The results indicated that TGEV could bind and activate the specific receptor EGFR in porcine intestinal epithelial cells after infection, leading to phosphorylation of ERK and regulation of the expression and activity NHE3, which resulted in dysfunctional NHE3 transport in intestinal epithelial cells. Extracellular Na^+^ could not flow into the cells because of continuous accumulation of an Na^+^ concentration gradient and the absorption of water and electrolyte-generated disorders.

NHE3 is mainly found in the brush border membrane of intestinal epithelial cells. The brush border consists of a large number of long microvilli. The number of NHE3 on the brush border membrane and the mobility of NHE3 on microvilli were significantly decreased under diarrheal conditions (Cha et al., 2010; Lin et al., 2010). Researchers found that LPA could promote the rapid movement of NHE3 from the bottom to the top of the microvillus to facilitate Na^+^ absorption, promoting the absorption of water-electrolytes and relieving the symptoms of diarrhea. The main mechanism of this regulation is that PKCδ-dependent LPA could recognize LPA5R to activate EGFR and ERK, resulting in their phosphorylation. NHE3 is released from the bottom of the microvilli to the top by PKCδ, which would enhance NHE3 mobility on brush border membrane (Yoo et al., 2011; Cha et al., 2014).

We used FRAP to study whether the mobility of NHE3 on the plasma membrane changes in intestinal epithelial cells after TGEV infection. The results showed that the mobile fraction of pEGFP-NHE3 in the uninfected cells was the highest and had the strongest mobility. We inferred that NHE3 is very mobile on the microvillus of the brush border membrane of small intestinal epithelial cells under normal physiological conditions. Continuous Na^+^ absorption is accomplished through NHE3 at the top of the plasma membrane by way of the lateral movement of the brush border membrane.

The mobile fractions of pEGFP-NHE3 decreased obviously in TGEV-infected cells, which might be due to the atrophy and rupture of intestinal epithelial microvillus in the small intestine, which would decrease the abundance and mobility of NHE3 on the brush border membrane. Compared with that in the TGEV-infected cells, NHE3 mobility in the TGEV-infected cells after EGFR inhibition was significantly increased. This result suggested that inhibition of EGFR activity could attenuate the damage to the brush border membrane microvilli caused by TGEV invasion and reduce intracellular virus proliferation in intestinal epithelial cells. In addition, NHE3 activity would be enhanced on the brush border membrane. Therefore, we speculated that the intensive mobility of NHE3 on the plasma membrane is beneficial for Na^+^ absorption in porcine intestinal epithelial cells. This speculation needs to be proven in further experiments.

**FIGURE 10:**
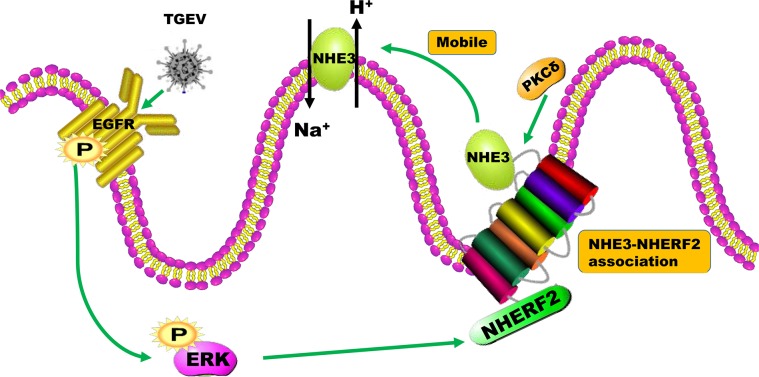
The mechanism of the regulation of NHE3 activity by the EGFR/ERK signaling pathway in intestinal epidermal cells after TGEV infection.

Based on the above results, the present study revealed that the activity and mobility of NHE3, regulated through the EGFR/ERK pathway on the brush border membrane of small intestinal epithelial cells, decreased after TGEV infection (Figure 10). This decreased the Na^+^/H^+^ transport mediated by NHE3 on the brush border membrane. Furthermore, the inhibition of EGFR was beneficial to the recovery of Na^+^ absorption in TGEV-infected cells.

## Author Contributions

ZY, YY, and ZS conceived and designed the experiments. ZY, LR, YY, KW, and PY performed the experiments. ZY, LX, SH, and JL analyzed the data. ZY, LR, and ZS wrote the manuscript.

## Conflict of Interest Statement

The authors declare that the research was conducted in the absence of any commercial or financial relationships that could be construed as a potential conflict of interest.
